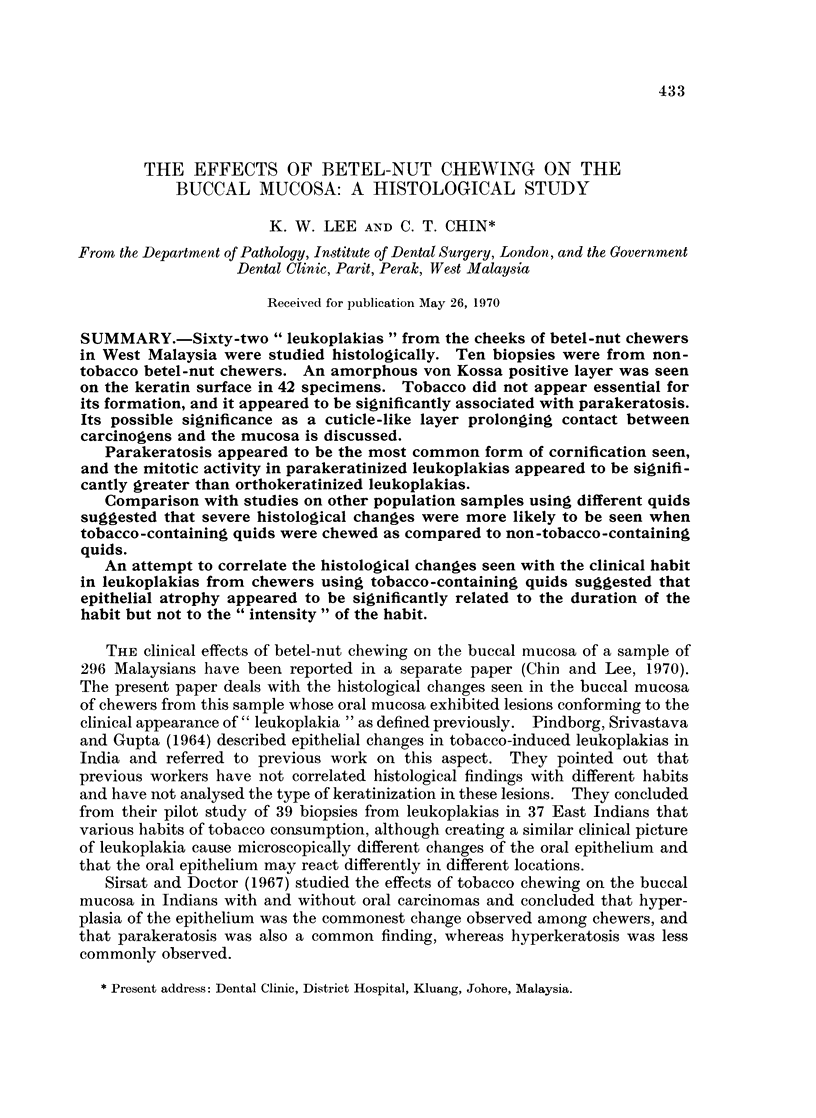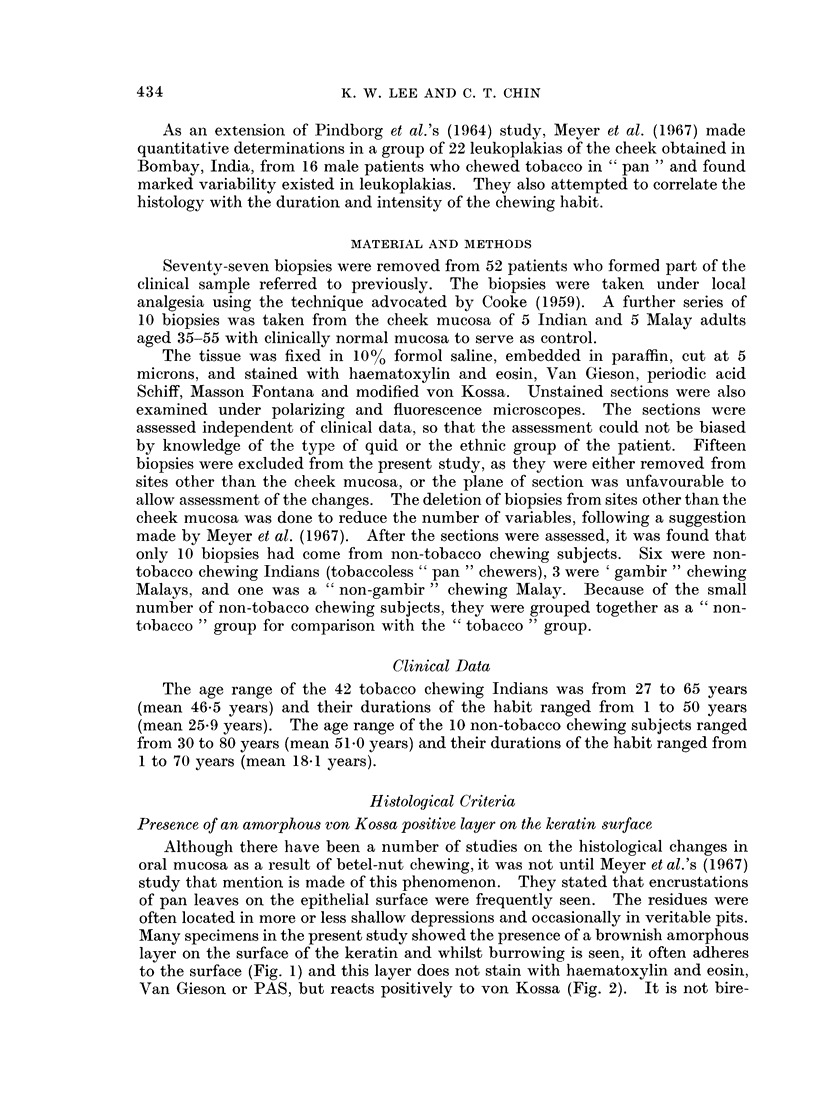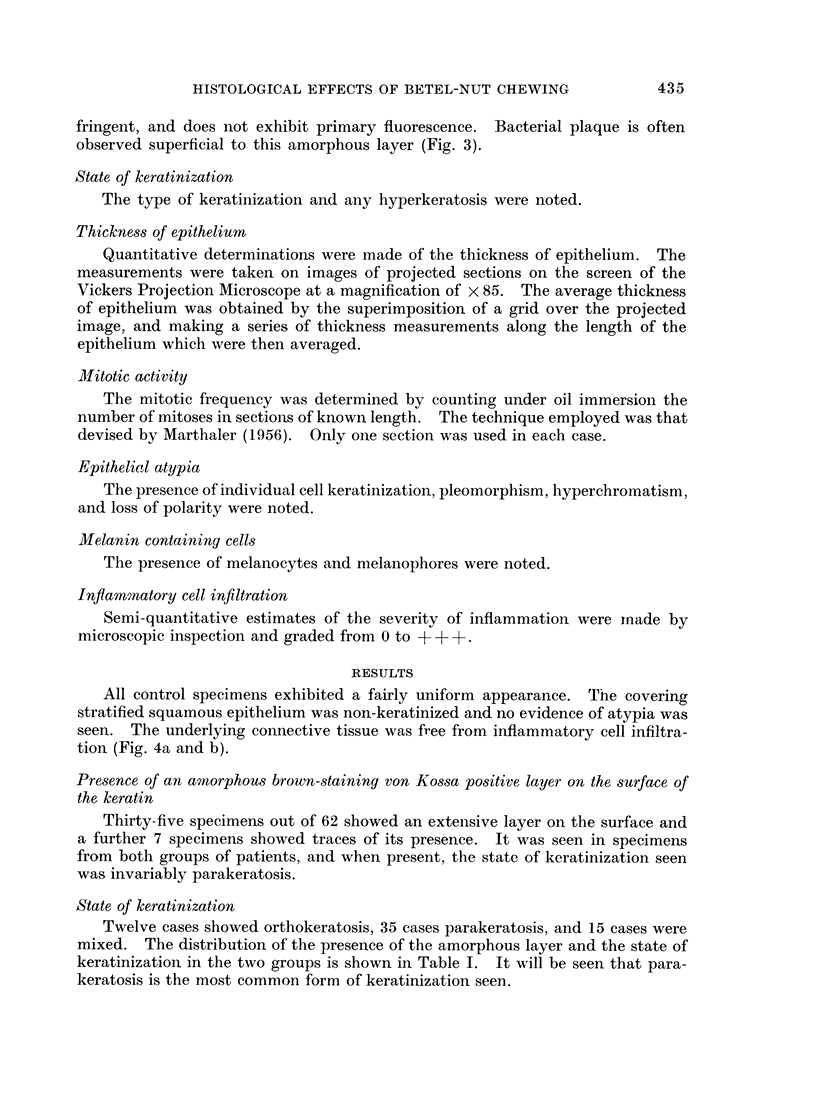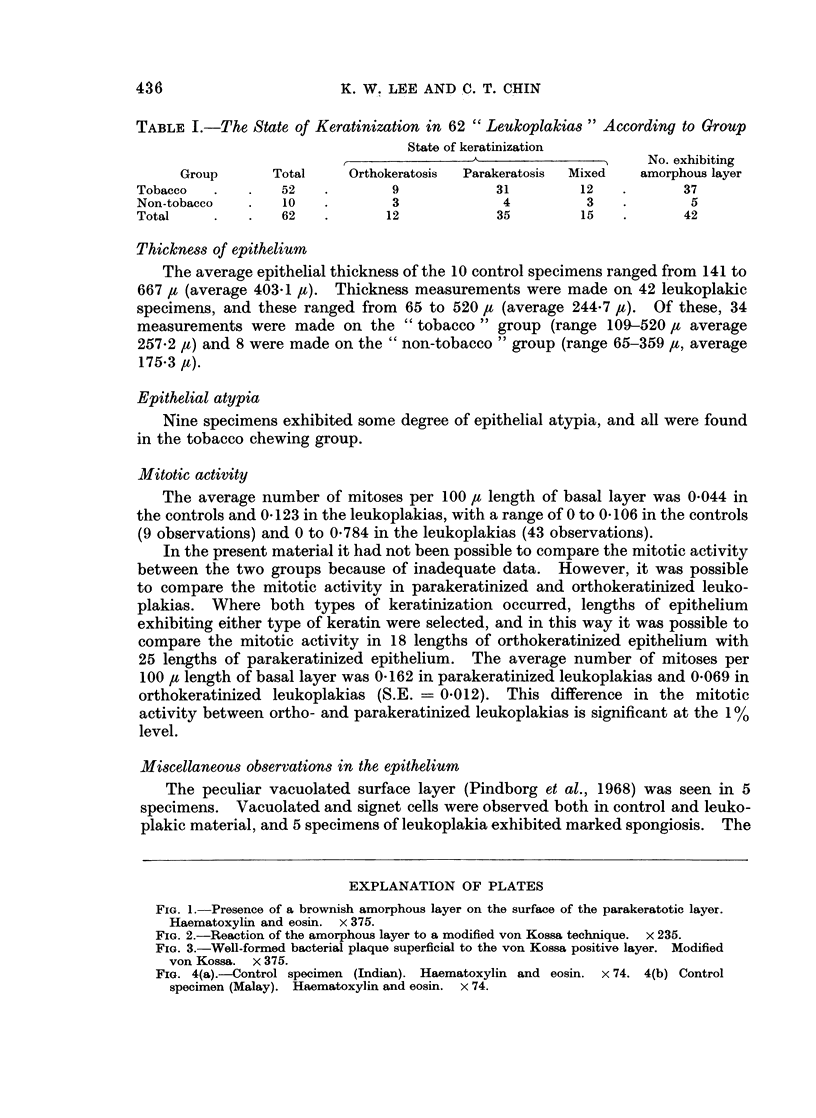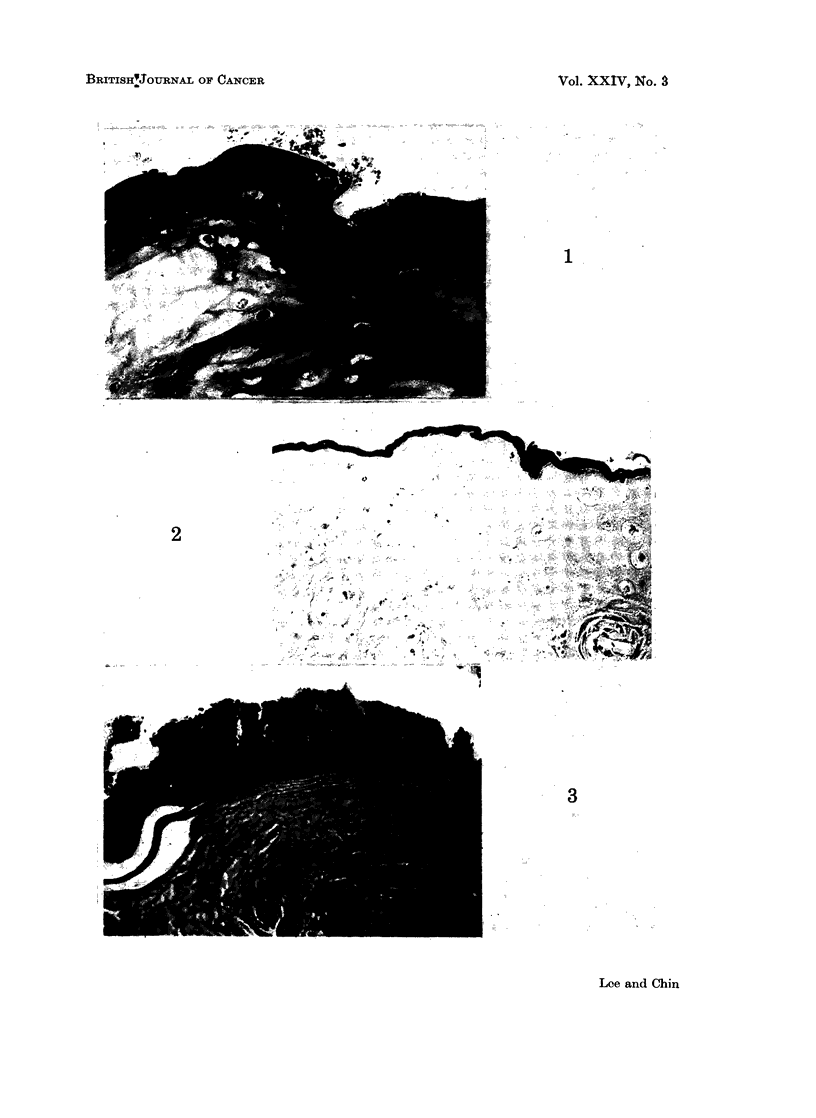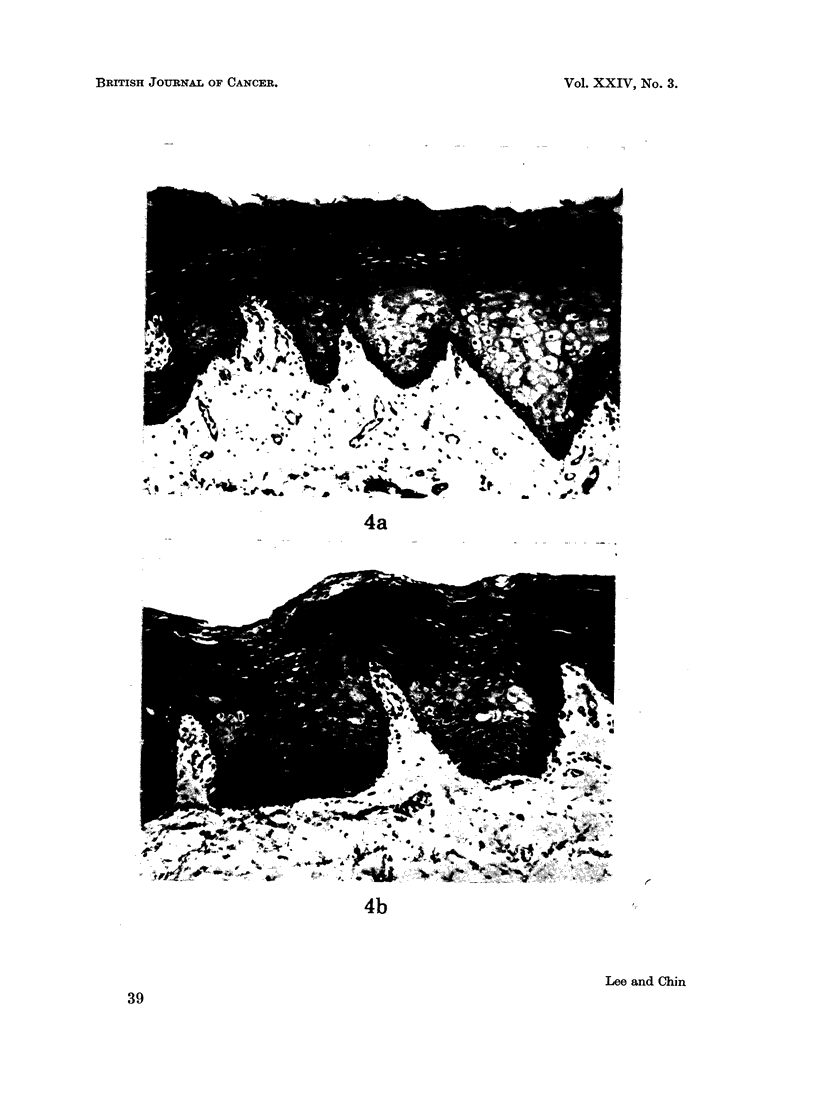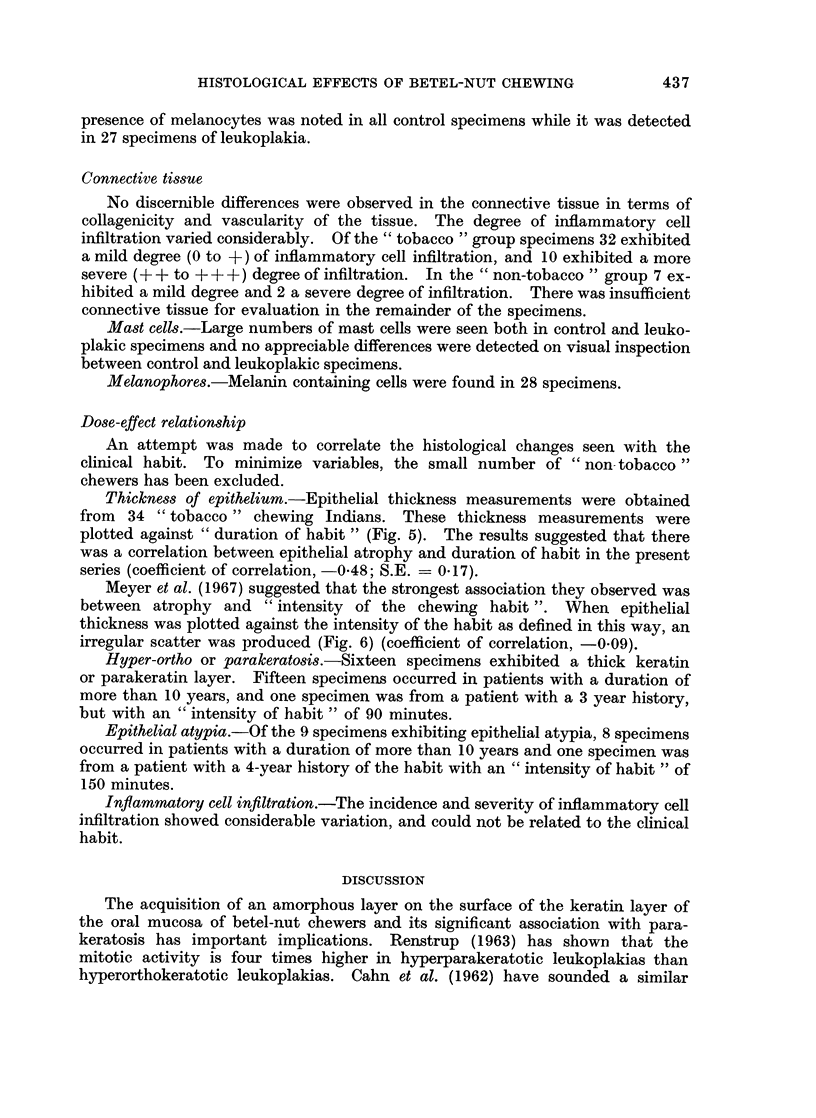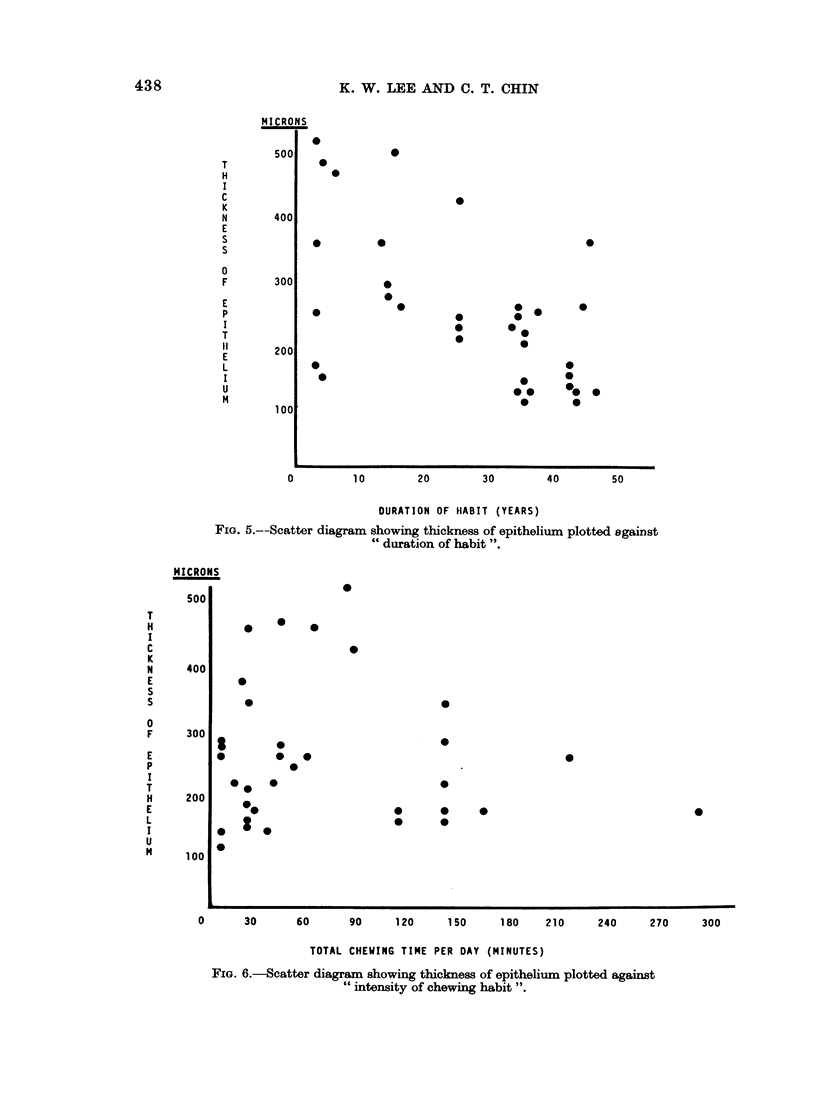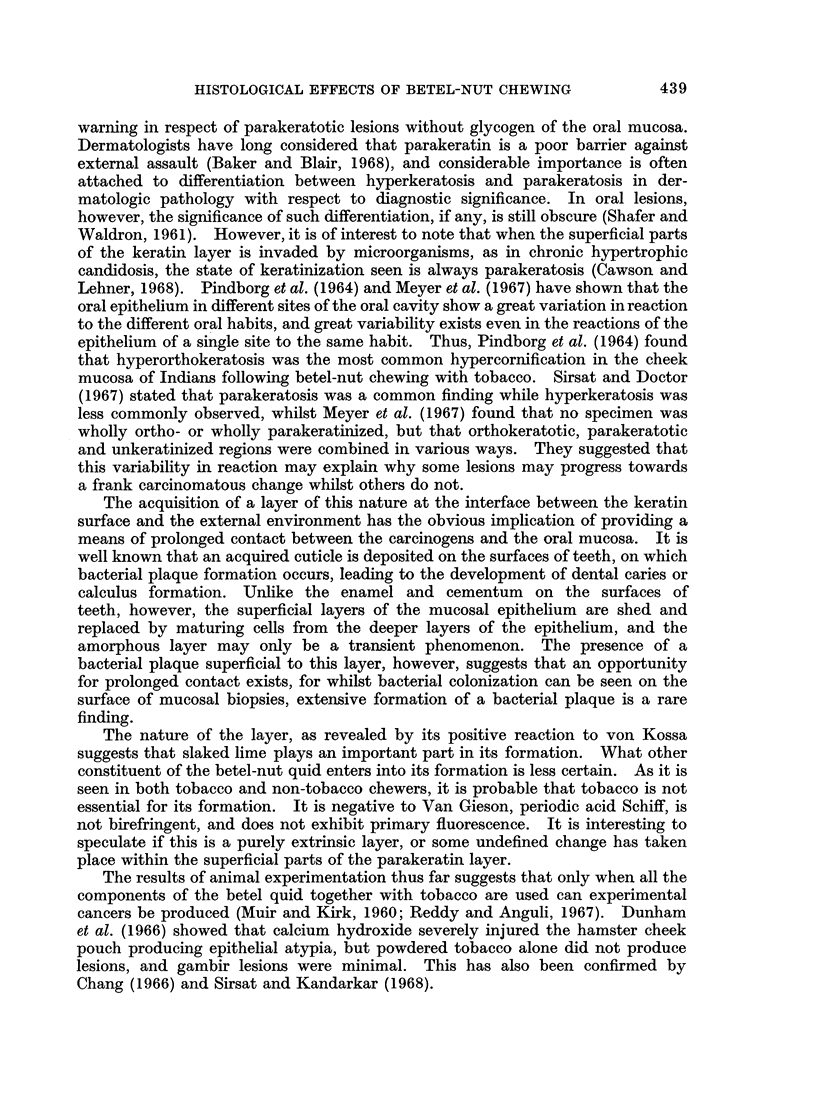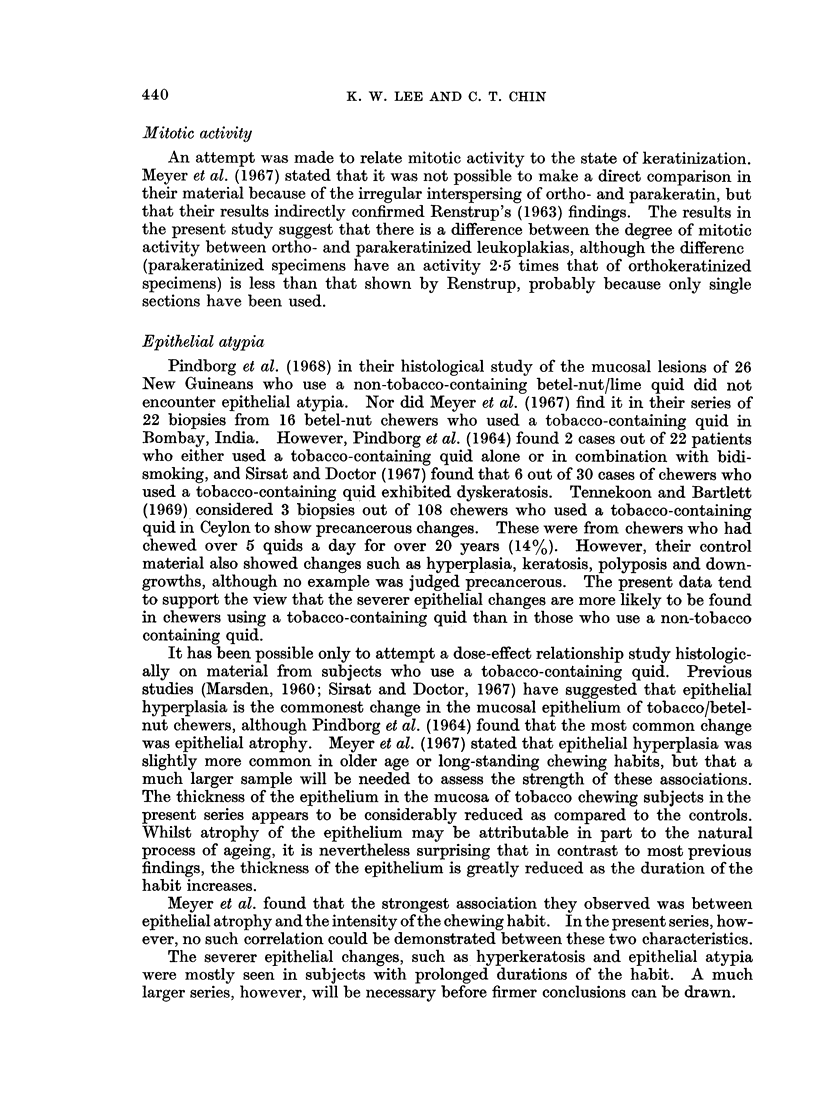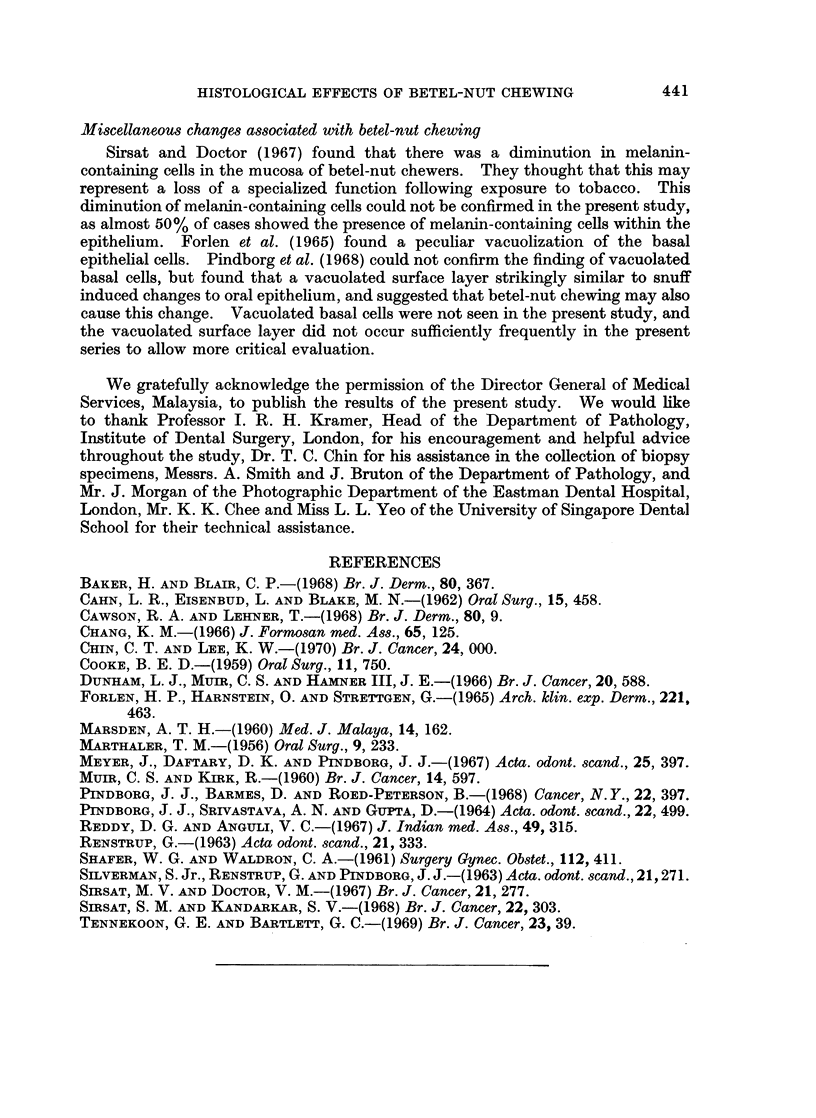# The Effects of Betel-Nut Chewing on the Buccal Mucosa: A Histological Study

**DOI:** 10.1038/bjc.1970.51

**Published:** 1970-09

**Authors:** K. W. Lee, C. T Chin

## Abstract

**Images:**


### THE EFFECTS OF BETEL-NUT CHEWING ON THE BUCCAL MUCOSA: A HISTOLOGICAL STUDY

K. W. LEE AND C. T. CHIN*

*From the Department of Pathology, Institute of Dental Surgery, London, and the Government Dental Clinic, Parit, Perak, West Malaysia*

Received for publication May 26, 1970

**SUMMARY.—Sixty-two " leukoplakias " from the cheeks of betel-nut chewers in West Malaysia were studied histologically. Ten biopsies were from non-tobacco betel-nut chewers. An amorphous von Kossa positive layer was seen on the keratin surface in 42 specimens. Tobacco did not appear essential for its formation, and it appeared to be significantly associated with parakeratosis. Its possible significance as a cuticle-like layer prolonging contact between carcinogens and the mucosa is discussed.**

**Parakeratosis appeared to be the most common form of cornification seen, and the mitotic activity in parakeratinized leukoplakias appeared to be significantly greater than orthokeratinized leukoplakias.**

**Comparison with studies on other population samples using different quids suggested that severe histological changes were more likely to be seen when tobacco-containing quids were chewed as compared to non-tobacco-containing quids.**

**An attempt to correlate the histological changes seen with the clinical habit in leukoplakias from chewers using tobacco-containing quids suggested that epithelial atrophy appeared to be significantly related to the duration of the habit but not to the " intensity " of the habit.**

THE clinical effects of betel-nut chewing on the buccal mucosa of a sample of 296 Malaysians have been reported in a separate paper (Chin and Lee, 1970). The present paper deals with the histological changes seen in the buccal mucosa of chewers from this sample whose oral mucosa exhibited lesions conforming to the clinical appearance of " leukoplakia " as defined previously. Pindborg, Srivastava and Gupta (1964) described epithelial changes in tobacco-induced leukoplakias in India and referred to previous work on this aspect. They pointed out that previous workers have not correlated histological findings with different habits and have not analysed the type of keratinization in these lesions. They concluded from their pilot study of 39 biopsies from leukoplakias in 37 East Indians that various habits of tobacco consumption, although creating a similar clinical picture of leukoplakia cause microscopically different changes of the oral epithelium and that the oral epithelium may react differently in different locations.

Sirsat and Doctor (1967) studied the effects of tobacco chewing on the buccal mucosa in Indians with and without oral carcinomas and concluded that hyperplasia of the epithelium was the commonest change observed among chewers, and that parakeratosis was also a common finding, whereas hyperkeratosis was less commonly observed.

* Present address: Dental Clinic, District Hospital, Kluang, Johore, Malaysia.

As an extension of Pindborg *et al.*'s (1964) study, Meyer *et al.* (1967) made quantitative determinations in a group of 22 leukoplakias of the cheek obtained in Bombay, India, from 16 male patients who chewed tobacco in " pan " and found marked variability existed in leukoplakias. They also attempted to correlate the histology with the duration and intensity of the chewing habit.

### MATERIAL AND METHODS

Seventy-seven biopsies were removed from 52 patients who formed part of the clinical sample referred to previously. The biopsies were taken under local analgesia using the technique advocated by Cooke (1959). A further series of 10 biopsies was taken from the cheek mucosa of 5 Indian and 5 Malay adults aged 35–55 with clinically normal mucosa to serve as control.

The tissue was fixed in 10% formol saline, embedded in paraffin, cut at 5 microns, and stained with haematoxylin and eosin, Van Gieson, periodic acid Schiff, Masson Fontana and modified von Kossa. Unstained sections were also examined under polarizing and fluorescence microscopes. The sections were assessed independent of clinical data, so that the assessment could not be biased by knowledge of the type of quid or the ethnic group of the patient. Fifteen biopsies were excluded from the present study, as they were either removed from sites other than the cheek mucosa, or the plane of section was unfavourable to allow assessment of the changes. The deletion of biopsies from sites other than the cheek mucosa was done to reduce the number of variables, following a suggestion made by Meyer *et al.* (1967). After the sections were assessed, it was found that only 10 biopsies had come from non-tobacco chewing subjects. Six were non-tobacco chewing Indians (tobaccoless " pan " chewers), 3 were ' gambir " chewing Malays, and one was a " non-gambir " chewing Malay. Because of the small number of non-tobacco chewing subjects, they were grouped together as a " non-tobacco " group for comparison with the " tobacco " group.

### *Clinical Data*

The age range of the 42 tobacco chewing Indians was from 27 to 65 years (mean 46·5 years) and their durations of the habit ranged from 1 to 50 years (mean 25·9 years). The age range of the 10 non-tobacco chewing subjects ranged from 30 to 80 years (mean 51·0 years) and their durations of the habit ranged from 1 to 70 years (mean 18·1 years).

### *Histological Criteria*

*Presence of an amorphous von Kossa positive layer on the keratin surface*

Although there have been a number of studies on the histological changes in oral mucosa as a result of betel-nut chewing, it was not until Meyer *et al.*'s (1967) study that mention is made of this phenomenon. They stated that encrustations of pan leaves on the epithelial surface were frequently seen. The residues were often located in more or less shallow depressions and occasionally in veritable pits. Many specimens in the present study showed the presence of a brownish amorphous layer on the surface of the keratin and whilst burrowing is seen, it often adheres to the surface (Fig. 1) and this layer does not stain with haematoxylin and eosin, Van Gieson or PAS, but reacts positively to von Kossa (Fig. 2). It is not birefringent, and does not exhibit primary fluorescence. Bacterial plaque is often observed superficial to this amorphous layer (Fig. 3).

*State of keratinization*

The type of keratinization and any hyperkeratosis were noted.

*Thickness of epithelium*

Quantitative determinations were made of the thickness of epithelium. The measurements were taken on images of projected sections on the screen of the Vickers Projection Microscope at a magnification of $\times 85$. The average thickness of epithelium was obtained by the superimposition of a grid over the projected image, and making a series of thickness measurements along the length of the epithelium which were then averaged.

*Mitotic activity*

The mitotic frequency was determined by counting under oil immersion the number of mitoses in sections of known length. The technique employed was that devised by Marthaler (1956). Only one section was used in each case.

*Epithelial atypia*

The presence of individual cell keratinization, pleomorphism, hyperchromatism, and loss of polarity were noted.

*Melanin containing cells*

The presence of melanocytes and melanophores were noted.

*Inflammatory cell infiltration*

Semi-quantitative estimates of the severity of inflammation were made by microscopic inspection and graded from 0 to $+++$.

### RESULTS

All control specimens exhibited a fairly uniform appearance. The covering stratified squamous epithelium was non-keratinized and no evidence of atypia was seen. The underlying connective tissue was free from inflammatory cell infiltration (Fig. 4a and b).

*Presence of an amorphous brown-staining von Kossa positive layer on the surface of the keratin*

Thirty-five specimens out of 62 showed an extensive layer on the surface and a further 7 specimens showed traces of its presence. It was seen in specimens from both groups of patients, and when present, the state of keratinization seen was invariably parakeratosis.

*State of keratinization*

Twelve cases showed orthokeratosis, 35 cases parakeratosis, and 15 cases were mixed. The distribution of the presence of the amorphous layer and the state of keratinization in the two groups is shown in Table I. It will be seen that parakeratosis is the most common form of keratinization seen.

TABLE I.—*The State of Keratinization in 62 " Leukoplakias " According to Group*

| Group | Total | State of keratinization: Orthokeratosis | Parakeratosis | Mixed | No. exhibiting amorphous layer |
|---|---|---|---|---|---|
| Tobacco | 52 | 9 | 31 | 12 | 37 |
| Non-tobacco | 10 | 3 | 4 | 3 | 5 |
| Total | 62 | 12 | 35 | 15 | 42 |

*Thickness of epithelium*

The average epithelial thickness of the 10 control specimens ranged from 141 to 667 $\mu$ (average 403·1 $\mu$). Thickness measurements were made on 42 leukoplakic specimens, and these ranged from 65 to 520 $\mu$ (average 244·7 $\mu$). Of these, 34 measurements were made on the " tobacco " group (range 109–520 $\mu$ average 257·2 $\mu$) and 8 were made on the " non-tobacco " group (range 65–359 $\mu$, average 175·3 $\mu$).

*Epithelial atypia*

Nine specimens exhibited some degree of epithelial atypia, and all were found in the tobacco chewing group.

*Mitotic activity*

The average number of mitoses per 100 $\mu$ length of basal layer was 0·044 in the controls and 0·123 in the leukoplakias, with a range of 0 to 0·106 in the controls (9 observations) and 0 to 0·784 in the leukoplakias (43 observations).

In the present material it had not been possible to compare the mitotic activity between the two groups because of inadequate data. However, it was possible to compare the mitotic activity in parakeratinized and orthokeratinized leukoplakias. Where both types of keratinization occurred, lengths of epithelium exhibiting either type of keratin were selected, and in this way it was possible to compare the mitotic activity in 18 lengths of orthokeratinized epithelium with 25 lengths of parakeratinized epithelium. The average number of mitoses per 100 $\mu$ length of basal layer was 0·162 in parakeratinized leukoplakias and 0·069 in orthokeratinized leukoplakias (S.E. = 0·012). This difference in the mitotic activity between ortho- and parakeratinized leukoplakias is significant at the 1% level.

*Miscellaneous observations in the epithelium*

The peculiar vacuolated surface layer (Pindborg *et al.*, 1968) was seen in 5 specimens. Vacuolated and signet cells were observed both in control and leukoplakic material, and 5 specimens of leukoplakia exhibited marked spongiosis. The

---

EXPLANATION OF PLATES

FIG. 1.—Presence of a brownish amorphous layer on the surface of the parakeratotic layer. Haematoxylin and eosin. ×375.

FIG. 2.—Reaction of the amorphous layer to a modified von Kossa technique. ×235.

FIG. 3.—Well-formed bacterial plaque superficial to the von Kossa positive layer. Modified von Kossa. ×375.

FIG. 4(a).—Control specimen (Indian). Haematoxylin and eosin. ×74. 4(b) Control specimen (Malay). Haematoxylin and eosin. ×74.

1

2

3

4a

4b

Lee and Chin presence of melanocytes was noted in all control specimens while it was detected in 27 specimens of leukoplakia.

*Connective tissue*

No discernible differences were observed in the connective tissue in terms of collagenicity and vascularity of the tissue. The degree of inflammatory cell infiltration varied considerably. Of the " tobacco " group specimens 32 exhibited a mild degree (0 to +) of inflammatory cell infiltration, and 10 exhibited a more severe (++ to +++) degree of infiltration. In the " non-tobacco " group 7 exhibited a mild degree and 2 a severe degree of infiltration. There was insufficient connective tissue for evaluation in the remainder of the specimens.

*Mast cells.*—Large numbers of mast cells were seen both in control and leukoplakic specimens and no appreciable differences were detected on visual inspection between control and leukoplakic specimens.

*Melanophores.*—Melanin containing cells were found in 28 specimens.

*Dose-effect relationship*

An attempt was made to correlate the histological changes seen with the clinical habit. To minimize variables, the small number of " non-tobacco " chewers has been excluded.

*Thickness of epithelium.*—Epithelial thickness measurements were obtained from 34 " tobacco " chewing Indians. These thickness measurements were plotted against " duration of habit " (Fig. 5). The results suggested that there was a correlation between epithelial atrophy and duration of habit in the present series (coefficient of correlation, —0·48; S.E. = 0·17).

Meyer *et al.* (1967) suggested that the strongest association they observed was between atrophy and " intensity of the chewing habit ". When epithelial thickness was plotted against the intensity of the habit as defined in this way, an irregular scatter was produced (Fig. 6) (coefficient of correlation, —0·09).

*Hyper-ortho* or *parakeratosis.*—Sixteen specimens exhibited a thick keratin or parakeratin layer. Fifteen specimens occurred in patients with a duration of more than 10 years, and one specimen was from a patient with a 3 year history, but with an " intensity of habit " of 90 minutes.

*Epithelial atypia.*—Of the 9 specimens exhibiting epithelial atypia, 8 specimens occurred in patients with a duration of more than 10 years and one specimen was from a patient with a 4-year history of the habit with an " intensity of habit " of 150 minutes.

*Inflammatory cell infiltration.*—The incidence and severity of inflammatory cell infiltration showed considerable variation, and could not be related to the clinical habit.

### DISCUSSION

The acquisition of an amorphous layer on the surface of the keratin layer of the oral mucosa of betel-nut chewers and its significant association with parakeratosis has important implications. Renstrup (1963) has shown that the mitotic activity is four times higher in hyperparakeratotic leukoplakias than hyperorthokeratotic leukoplakias. Cahn *et al.* (1962) have sounded a similar

Fig. 5.—Scatter diagram showing thickness of epithelium plotted against " duration of habit ".

Fig. 6.—Scatter diagram showing thickness of epithelium plotted against " intensity of chewing habit ".

warning in respect of parakeratotic lesions without glycogen of the oral mucosa. Dermatologists have long considered that parakeratin is a poor barrier against external assault (Baker and Blair, 1968), and considerable importance is often attached to differentiation between hyperkeratosis and parakeratosis in dermatologic pathology with respect to diagnostic significance. In oral lesions, however, the significance of such differentiation, if any, is still obscure (Shafer and Waldron, 1961). However, it is of interest to note that when the superficial parts of the keratin layer is invaded by microorganisms, as in chronic hypertrophic candidosis, the state of keratinization seen is always parakeratosis (Cawson and Lehner, 1968). Pindborg *et al.* (1964) and Meyer *et al.* (1967) have shown that the oral epithelium in different sites of the oral cavity show a great variation in reaction to the different oral habits, and great variability exists even in the reactions of the epithelium of a single site to the same habit. Thus, Pindborg *et al.* (1964) found that hyperorthokeratosis was the most common hypercornification in the cheek mucosa of Indians following betel-nut chewing with tobacco. Sirsat and Doctor (1967) stated that parakeratosis was a common finding while hyperkeratosis was less commonly observed, whilst Meyer *et al.* (1967) found that no specimen was wholly ortho- or wholly parakeratinized, but that orthokeratotic, parakeratotic and unkeratinized regions were combined in various ways. They suggested that this variability in reaction may explain why some lesions may progress towards a frank carcinomatous change whilst others do not.

The acquisition of a layer of this nature at the interface between the keratin surface and the external environment has the obvious implication of providing a means of prolonged contact between the carcinogens and the oral mucosa. It is well known that an acquired cuticle is deposited on the surfaces of teeth, on which bacterial plaque formation occurs, leading to the development of dental caries or calculus formation. Unlike the enamel and cementum on the surfaces of teeth, however, the superficial layers of the mucosal epithelium are shed and replaced by maturing cells from the deeper layers of the epithelium, and the amorphous layer may only be a transient phenomenon. The presence of a bacterial plaque superficial to this layer, however, suggests that an opportunity for prolonged contact exists, for whilst bacterial colonization can be seen on the surface of mucosal biopsies, extensive formation of a bacterial plaque is a rare finding.

The nature of the layer, as revealed by its positive reaction to von Kossa suggests that slaked lime plays an important part in its formation. What other constituent of the betel-nut quid enters into its formation is less certain. As it is seen in both tobacco and non-tobacco chewers, it is probable that tobacco is not essential for its formation. It is negative to Van Gieson, periodic acid Schiff, is not birefringent, and does not exhibit primary fluorescence. It is interesting to speculate if this is a purely extrinsic layer, or some undefined change has taken place within the superficial parts of the parakeratin layer.

The results of animal experimentation thus far suggests that only when all the components of the betel quid together with tobacco are used can experimental cancers be produced (Muir and Kirk, 1960; Reddy and Anguli, 1967). Dunham *et al.* (1966) showed that calcium hydroxide severely injured the hamster cheek pouch producing epithelial atypia, but powdered tobacco alone did not produce lesions, and gambir lesions were minimal. This has also been confirmed by Chang (1966) and Sirsat and Kandarkar (1968).

*Mitotic activity*

An attempt was made to relate mitotic activity to the state of keratinization. Meyer *et al.* (1967) stated that it was not possible to make a direct comparison in their material because of the irregular interspersing of ortho- and parakeratin, but that their results indirectly confirmed Renstrup's (1963) findings. The results in the present study suggest that there is a difference between the degree of mitotic activity between ortho- and parakeratinized leukoplakias, although the differenc (parakeratinized specimens have an activity 2·5 times that of orthokeratinized specimens) is less than that shown by Renstrup, probably because only single sections have been used.

*Epithelial atypia*

Pindborg *et al.* (1968) in their histological study of the mucosal lesions of 26 New Guineans who use a non-tobacco-containing betel-nut/lime quid did not encounter epithelial atypia. Nor did Meyer *et al.* (1967) find it in their series of 22 biopsies from 16 betel-nut chewers who used a tobacco-containing quid in Bombay, India. However, Pindborg *et al.* (1964) found 2 cases out of 22 patients who either used a tobacco-containing quid alone or in combination with bidi-smoking, and Sirsat and Doctor (1967) found that 6 out of 30 cases of chewers who used a tobacco-containing quid exhibited dyskeratosis. Tennekoon and Bartlett (1969) considered 3 biopsies out of 108 chewers who used a tobacco-containing quid in Ceylon to show precancerous changes. These were from chewers who had chewed over 5 quids a day for over 20 years (14%). However, their control material also showed changes such as hyperplasia, keratosis, polyposis and down-growths, although no example was judged precancerous. The present data tend to support the view that the severer epithelial changes are more likely to be found in chewers using a tobacco-containing quid than in those who use a non-tobacco containing quid.

It has been possible only to attempt a dose-effect relationship study histologically on material from subjects who use a tobacco-containing quid. Previous studies (Marsden, 1960; Sirsat and Doctor, 1967) have suggested that epithelial hyperplasia is the commonest change in the mucosal epithelium of tobacco/betel-nut chewers, although Pindborg *et al.* (1964) found that the most common change was epithelial atrophy. Meyer *et al.* (1967) stated that epithelial hyperplasia was slightly more common in older age or long-standing chewing habits, but that a much larger sample will be needed to assess the strength of these associations. The thickness of the epithelium in the mucosa of tobacco chewing subjects in the present series appears to be considerably reduced as compared to the controls. Whilst atrophy of the epithelium may be attributable in part to the natural process of ageing, it is nevertheless surprising that in contrast to most previous findings, the thickness of the epithelium is greatly reduced as the duration of the habit increases.

Meyer *et al.* found that the strongest association they observed was between epithelial atrophy and the intensity of the chewing habit. In the present series, however, no such correlation could be demonstrated between these two characteristics.

The severer epithelial changes, such as hyperkeratosis and epithelial atypia were mostly seen in subjects with prolonged durations of the habit. A much larger series, however, will be necessary before firmer conclusions can be drawn.

*Miscellaneous changes associated with betel-nut chewing*

Sirsat and Doctor (1967) found that there was a diminution in melanin-containing cells in the mucosa of betel-nut chewers. They thought that this may represent a loss of a specialized function following exposure to tobacco. This diminution of melanin-containing cells could not be confirmed in the present study, as almost 50% of cases showed the presence of melanin-containing cells within the epithelium. Forlen *et al.* (1965) found a peculiar vacuolization of the basal epithelial cells. Pindborg *et al.* (1968) could not confirm the finding of vacuolated basal cells, but found that a vacuolated surface layer strikingly similar to snuff induced changes to oral epithelium, and suggested that betel-nut chewing may also cause this change. Vacuolated basal cells were not seen in the present study, and the vacuolated surface layer did not occur sufficiently frequently in the present series to allow more critical evaluation.

We gratefully acknowledge the permission of the Director General of Medical Services, Malaysia, to publish the results of the present study. We would like to thank Professor I. R. H. Kramer, Head of the Department of Pathology, Institute of Dental Surgery, London, for his encouragement and helpful advice throughout the study, Dr. T. C. Chin for his assistance in the collection of biopsy specimens, Messrs. A. Smith and J. Bruton of the Department of Pathology, and Mr. J. Morgan of the Photographic Department of the Eastman Dental Hospital, London, Mr. K. K. Chee and Miss L. L. Yeo of the University of Singapore Dental School for their technical assistance.

### REFERENCES

Baker, H. and Blair, C. P.—(1968) *Br. J. Derm.*, **80**, 367.

Cahn, L. R., Eisenbud, L. and Blake, M. N.—(1962) *Oral Surg.*, **15**, 458.

Cawson, R. A. and Lehner, T.—(1968) *Br. J. Derm.*, **80**, 9.

Chang, K. M.—(1966) *J. Formosan med. Ass.*, **65**, 125.

Chin, C. T. and Lee, K. W.—(1970) *Br. J. Cancer*, **24**, 000.

Cooke, B. E. D.—(1959) *Oral Surg.*, **11**, 750.

Dunham, L. J., Muir, C. S. and Hamner III, J. E.—(1966) *Br. J. Cancer*, **20**, 588.

Forlen, H. P., Harnstein, O. and Strettgen, G.—(1965) *Arch. klin. exp. Derm.*, **221**, 463.

Marsden, A. T. H.—(1960) *Med. J. Malaya*, **14**, 162.

Marthaler, T. M.—(1956) *Oral Surg.*, **9**, 233.

Meyer, J., Daftary, D. K. and Pindborg, J. J.—(1967) *Acta. odont. scand.*, **25**, 397.

Muir, C. S. and Kirk, R.—(1960) *Br. J. Cancer*, **14**, 597.

Pindborg, J. J., Barmes, D. and Roed-Peterson, B.—(1968) *Cancer, N.Y.*, **22**, 397.

Pindborg, J. J., Srivastava, A. N. and Gupta, D.—(1964) *Acta. odont. scand.*, **22**, 499.

Reddy, D. G. and Anguli, V. C.—(1967) *J. Indian med. Ass.*, **49**, 315.

Renstrup, G.—(1963) *Acta odont. scand.*, **21**, 333.

Shafer, W. G. and Waldron, C. A.—(1961) *Surgery Gynec. Obstet.*, **112**, 411.

Silverman, S. Jr., Renstrup, G. and Pindborg, J. J.—(1963) *Acta. odont. scand.*, **21**, 271.

Sirsat, M. V. and Doctor, V. M.—(1967) *Br. J. Cancer*, **21**, 277.

Sirsat, S. M. and Kandarkar, S. V.—(1968) *Br. J. Cancer*, **22**, 303.

Tennekoon, G. E. and Bartlett, G. C.—(1969) *Br. J. Cancer*, **23**, 39.